# Bill of Mortality for Portsmouth, New Hampshire, for 1804

**Published:** 1807-01

**Authors:** Lyman Spalding


### *Bill of Mortality for Portsmouth, New Hampshire, for* 1804.
### By LYMAN SPALDING, M. D.

| | | | |
|---|---|---|---|
| Aphtha | 2 | Nonclosure of the Foramen Ovale and Canaliteriosus | 1 |
| Apoplexy | 2 | Old Age | 6 |
| Atrophy | 9 | Palsy | 5 |
| Cholera of Infants | 5 | Phrenzy | 1 |
| Consumption | 26 | Premature Birth | 3 |
| Convulsions | 13 | Quinsy | 4 |
| Dropsy | 3 | CASUALTIES. | |
| Dropsy in the Brain | 5 | Drowned | 2 |
| Dysentery | 1 | Fall | 3 |
| Fever Billious | 2 | Scalded | 2 |
| Fever Puerperal | 1 | BIRTHS. | |
| Fever Pulmonic | 3 | Males | 163 |
| Gravel | 1 | Females | 130 |
| Hooping Cough | 4 | Marriages | 64 |
| Lock Jaw | 1 | | |
| Mortification | 5 | | |

Portsmouth, the capital of the state of New Hampshire, situated 43°, 5′, north latitude, and 6°, 26′, east longitude from Washington, contains above 6000 inhabitants.

The cholera of infants made its appearance in June, and continued into November; the hooping cough appeared in September, and prevailed to the end of the year; the quinsy, or croup, was first noticed in October, and its footsteps are traced to the last of December.

The nonclosure of the foramen ovale, in a child of two days old, is a singular occurrence; the child was very healthy, strong and robust, but suddenly changed to a blue colour, gasping for breath; friction and warm bathing restored its colour but for a few minutes, the blue appearance returned, and he died gasping for breath, evidently for want of the circulation through the lungs.

As

As usual, the consumption is highest on the list; nearly one fourth part have died of that disease. How long shall this remain the opprobrium of the art! It is unusual for the consumption to appear so early in life as the 11th year, but this, in a lad of colour, was an unquestionable case of phthisis pulmonalis.

The town has exhibited its usual healthfulness this year, not one in 55 having died, differing essentially from the two last years. The births compared to the deaths are nearly as three to one.

